# Supplementary material for: Murine modeling of IDH-mutant 1p/19q-codeleted oligodendroglioma reveals genotype specific phenotypes
Source: bioRxiv. 2026 May 19:2026.05.14.725183. Preprint. [Version 2] doi: 10.64898/2026.05.14.725183 (PMC13228301; doi:10.64898/2026.05.14.725183)
Supplement: Supplement 1 [file NIHPP2026.05.14.725183v2-supplement-1.pdf]

bioRxiv preprint doi: https://doi.org/10.64898/2026.05.14.725183; this version posted May 19, 2026. The copyright holder for this preprint (which was not certified by peer review) is the author/funder, who has granted bioRxiv a license to display the preprint in perpetuity. It is made available under a CC-BY 4.0 International license.

Supplementary Figure 1

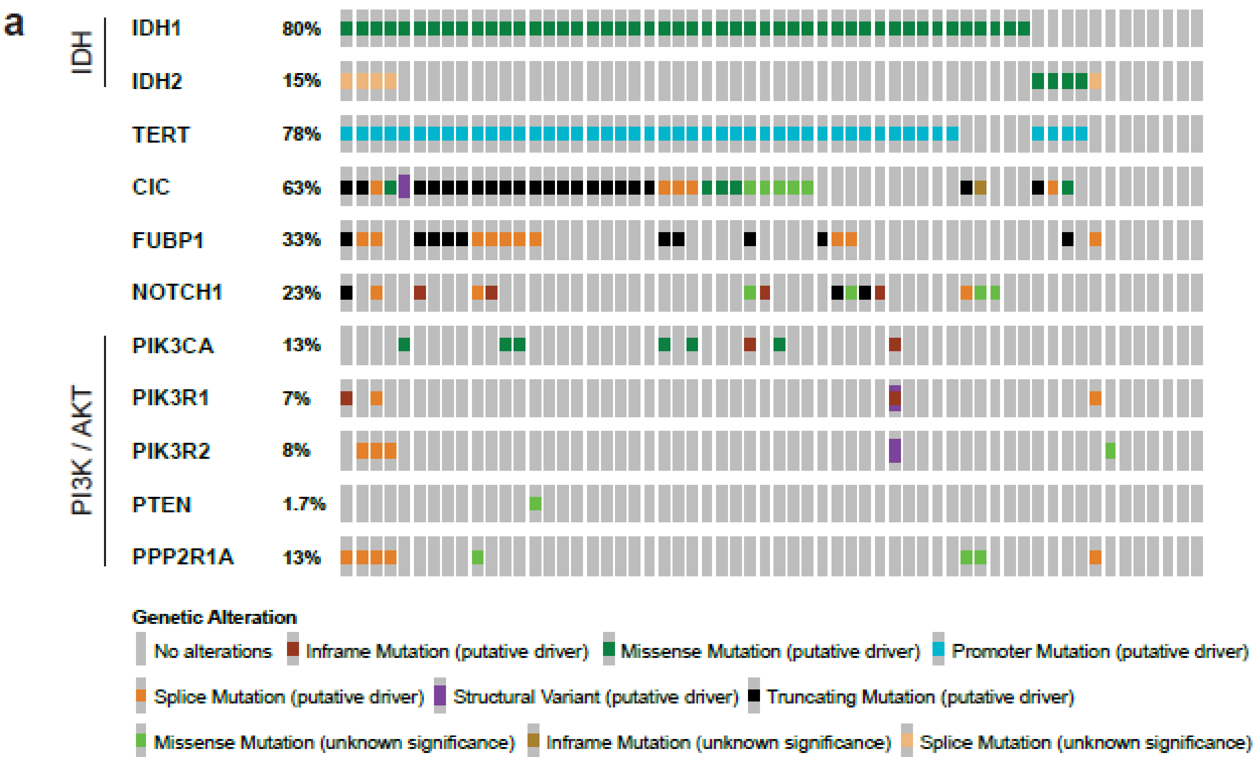

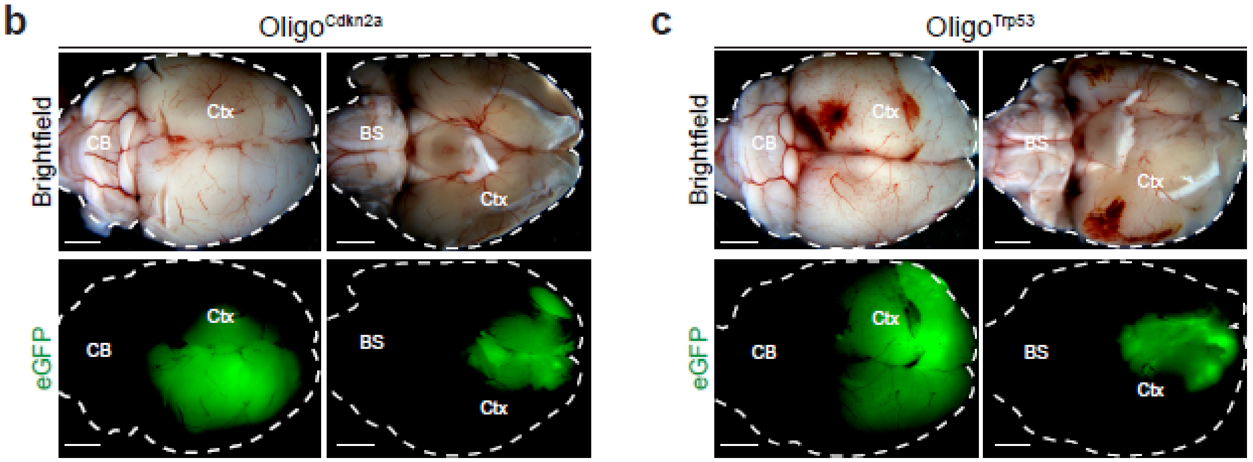

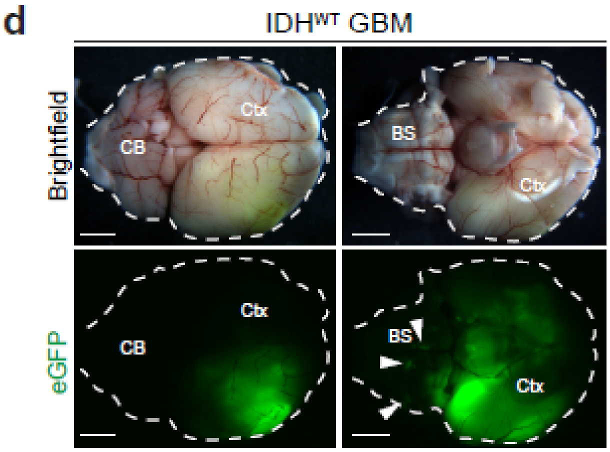

bioRxiv preprint doi: https://doi.org/10.64898/2026.05.14.725183; this version posted May 19, 2026. The copyright holder for this preprint (which was not certified by peer review) is the author/funder, who has granted bioRxiv a license to display the preprint in perpetuity. It is made available under a CC-BY 4.0 International license.

Supplementary Figure 1 – The genetic landscape of human IDH-mutant 1p/19q codeleted oligodendroglioma. a, Oncoprint displaying the top recurrent genetic alterations across 62 confirmed IDH-mutant 1p/19q oligodendroglioma patients (Oligo Nation/CBTN provision dataset). **b-d,** Brightfield and GFP whole brain images demonstrating the location of GFP-positive tumor cells across IUE conditions. Arrowheads denote spread of GFP-positive cells into the brainstem of IDH^{WT} GBM. Scale bar: 200µm. Ctx: cortex; CB: cerebellum; BS: brainstem.

bioRxiv preprint doi: https://doi.org/10.64898/2026.05.14.725183; this version posted May 19, 2026. The copyright holder for this preprint (which was not certified by peer review) is the author/funder, who has granted bioRxiv a license to display the preprint in perpetuity. It is made available under a CC-BY 4.0 International license.

Supplementary Figure 2

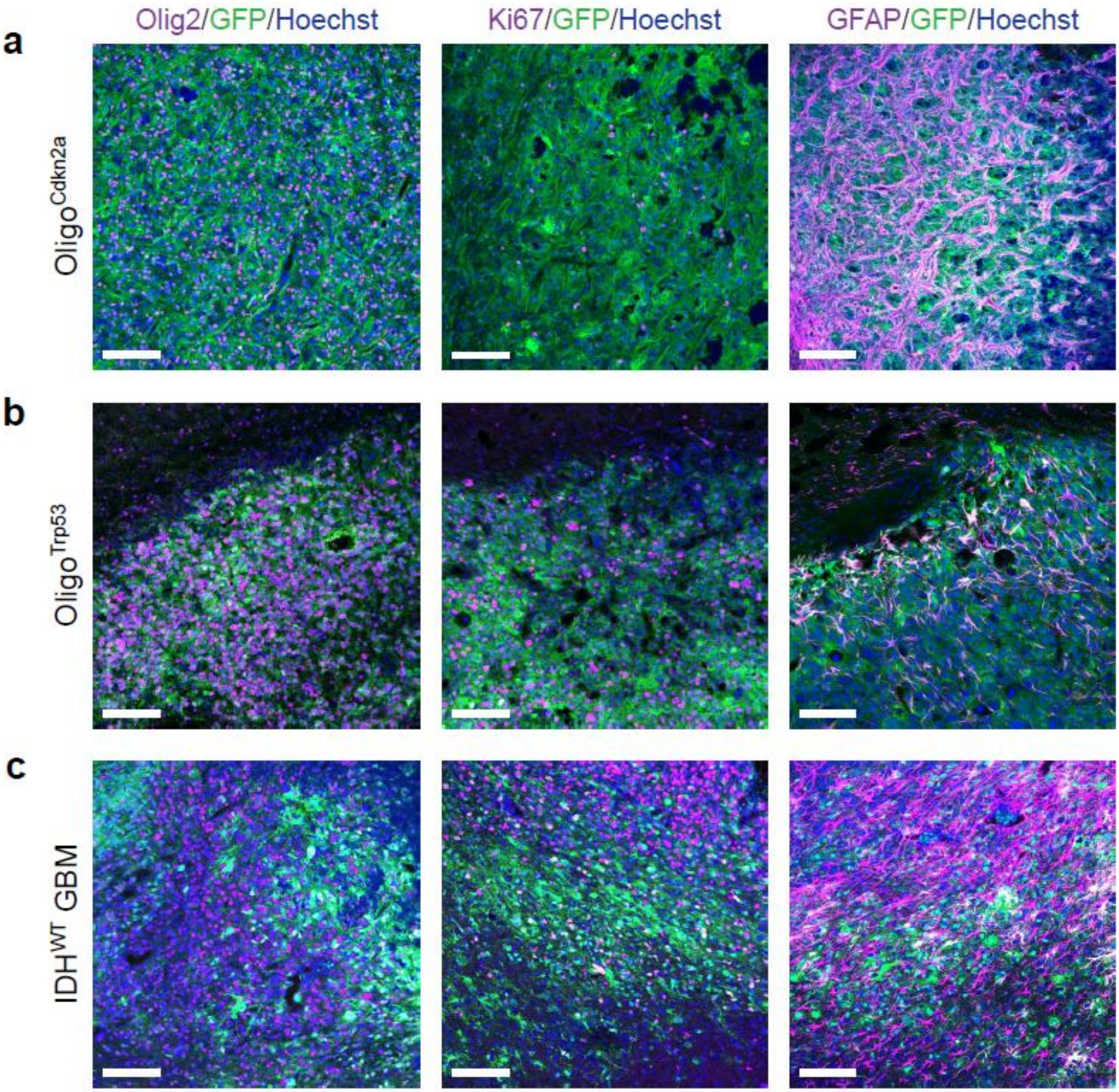

Supplementary Figure 2 – Shared expression of glioma markers in Oligo$^{Cdkn2a/}$, Oligo^{Trp53} and IDH^{WT} GBM mouse models. **a,** Oligo^{Cdkn2a}, **b,** Oligo^{Trp53}, and **c,** IDH^{WT} GBM tumor samples co-stained for GFP (green), Hoechst (blue) and either Olig2, Ki67 or GFAP (magenta). Scale bar: 100μm.

bioRxiv preprint doi: https://doi.org/10.64898/2026.05.14.725183; this version posted May 19, 2026. The copyright holder for this preprint (which was not certified by peer review) is the author/funder, who has granted bioRxiv a license to display the preprint in perpetuity. It is made available under a CC-BY 4.0 International license.

Supplementary Figure 3

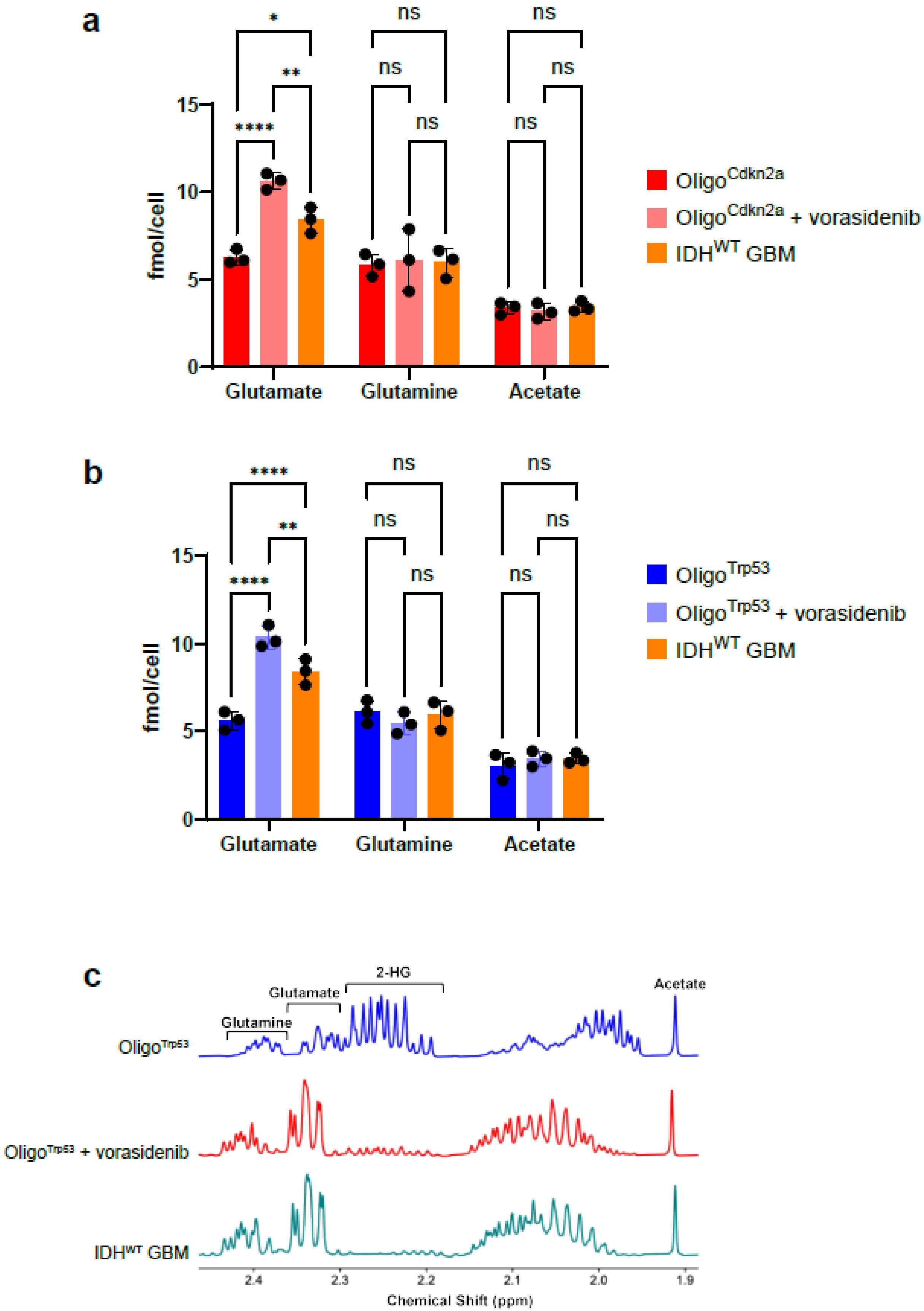

bioRxiv preprint doi: https://doi.org/10.64898/2026.05.14.725183; this version posted May 19, 2026. The copyright holder for this preprint (which was not certified by peer review) is the author/funder, who has granted bioRxiv a license to display the preprint in perpetuity. It is made available under a CC-BY 4.0 International license.

Supplementary Figure 3 – Metabolic profiling of oligodendroglioma models and response to IDH inhibition. a, Metabolite levels (fmol/cell) in Oligo^{Cdkn2a} cells treated with DMSO or vorasidenib (uM, 72 hrs) and IDH^{WT} GBM cells. **b,** Metabolite levels (fmol/cell) in Oligo^{Trp53} cells treated with DMSO or vorasidenib (uM, 72 hrs) and IDH^{WT} GBM cells. Values indicate mean ± SD; Two-way ANOVA with Sidak's multiple comparisons test. *p<0.05, **p<0.005, ***p<0.001, ****p<0.0001. Oligo^{Cdkn2a} n=3, Oligo^{Trp53} n=3, IDH^{WT} GBM n=3. **c,** Representative ^{1}H magnetic resonance spectra illustrating the metabolic profiles of Oligo^{Trp53} (top trace), vorasidenib-treated Oligo^{Trp53} (middle trace), and IDH^{WT} GBM (bottom trace) cell extracts. Peaks corresponding to glutamine, glutamate, 2-hydroxyglutarate (2-HG), and acetate are indicated across the 1.9–2.4 ppm chemical shift range.

bioRxiv preprint doi: https://doi.org/10.64898/2026.05.14.725183; this version posted May 19, 2026. The copyright holder for this preprint (which was not certified by peer review) is the author/funder, who has granted bioRxiv a license to display the preprint in perpetuity. It is made available under a CC-BY 4.0 International license.

Supplementary Figure 4

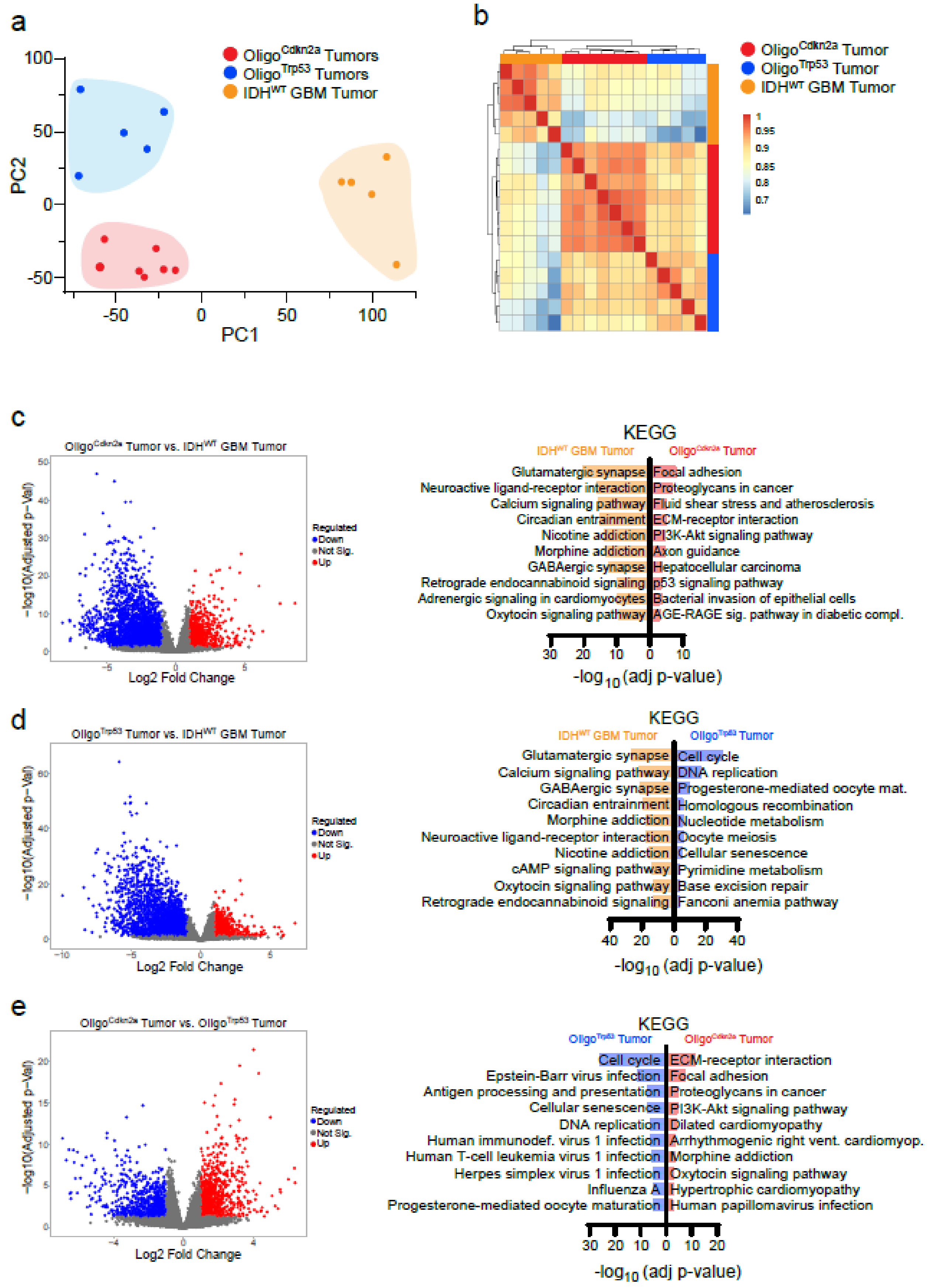

bioRxiv preprint doi: https://doi.org/10.64898/2026.05.14.725183; this version posted May 19, 2026. The copyright holder for this preprint (which was not certified by peer review) is the author/funder, who has granted bioRxiv a license to display the preprint in perpetuity. It is made available under a CC-BY 4.0 International license.

Supplementary Figure 4 – IDH- and Trp53-mutation status impact the transcriptional profile of tumor models. a, PCA plot of all tumor samples based on transcriptome-wide expression. **b,** Pearson's correlation plot of all tumor samples. **c,** Volcano plot displaying significantly up- and down-regulated genes between $Oligo^{Cdkn2a}$ vs IDH^{WT} GBM tumors, and ORA of KEGG gene sets in each condition. The top five most significant gene sets are plotted by $-log_{10}$(adj-pvalue). **d,** Volcano plot displaying significantly up- and down-regulated genes between $Oligo^{Trp53}$ vs IDH^{WT} GBM tumors, and ORA of KEGG gene sets in each condition. The top five most significant gene sets are plotted by $-log_{10}$(adj-pvalue). **e,** Volcano plot displaying significantly up- and down-regulated genes between $Oligo^{Cdkn2a}$ vs $Oligo^{Trp53}$ tumors, and ORA of KEGG gene sets in each condition. The top five most significant gene sets are plotted by $-log_{10}$(adj-pvalue).

bioRxiv preprint doi: https://doi.org/10.64898/2026.05.14.725183; this version posted May 19, 2026. The copyright holder for this preprint (which was not certified by peer review) is the author/funder, who has granted bioRxiv a license to display the preprint in perpetuity. It is made available under a CC-BY 4.0 International license.

Supplementary Figure 5

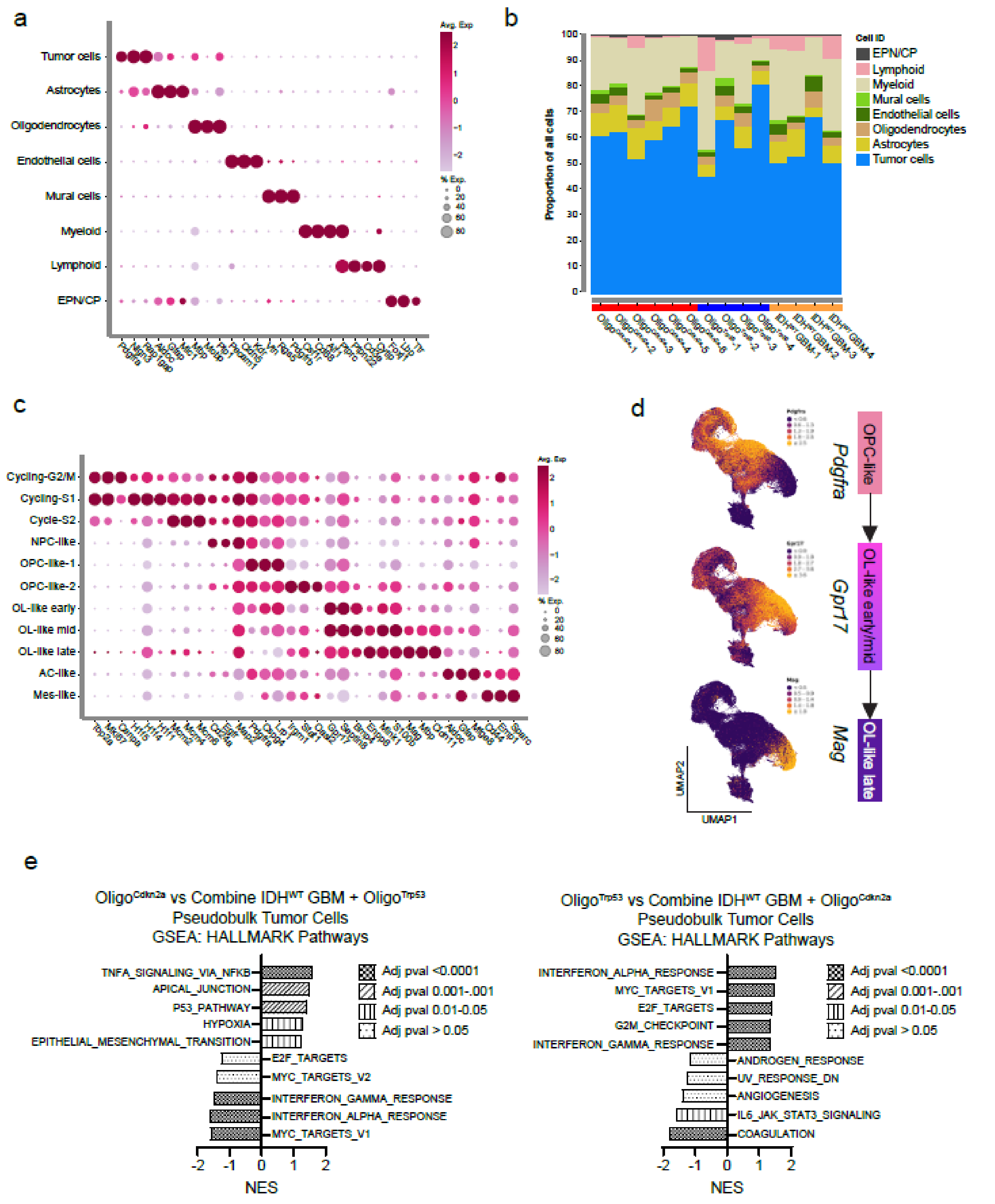

bioRxiv preprint doi: https://doi.org/10.64898/2026.05.14.725183; this version posted May 19, 2026. The copyright holder for this preprint (which was not certified by peer review) is the author/funder, who has granted bioRxiv a license to display the preprint in perpetuity. It is made available under a CC-BY 4.0 International license.

Supplementary Figure 5 – Single cell profiling identifies tumor cell-state and transcriptional heterogeneity driven by tumor genetics. **a,** Dot plot displaying the relative expression of cell-type specific markers in the designated cell-types. **b,** Stacked bar plot illustrating the relative proportion of immune cell populations in individual tumor samples. **c,** Dot plot displaying the relative expression of cell-type specific markers in the designated tumor cell-types. NPC: neural progenitor cell; OPC: oligodendrocyte progenitor cell; OL: oligodendroglioma; AC: astrocytic cell; Mes: mesenchymal. **d,** UMAP visualization of tumor cell clusters showing expression of selected oligodendrocyte lineage markers. **e,** GSEA of HALLMARK gene sets for pseudo bulk tumor cell comparison. The top five most significant (adj-pval) gene sets are plotted by normalized enrichment score (NES).

bioRxiv preprint doi: https://doi.org/10.64898/2026.05.14.725183; this version posted May 19, 2026. The copyright holder for this preprint (which was not certified by peer review) is the author/funder, who has granted bioRxiv a license to display the preprint in perpetuity. It is made available under a CC-BY 4.0 International license.

Supplementary Figure 6

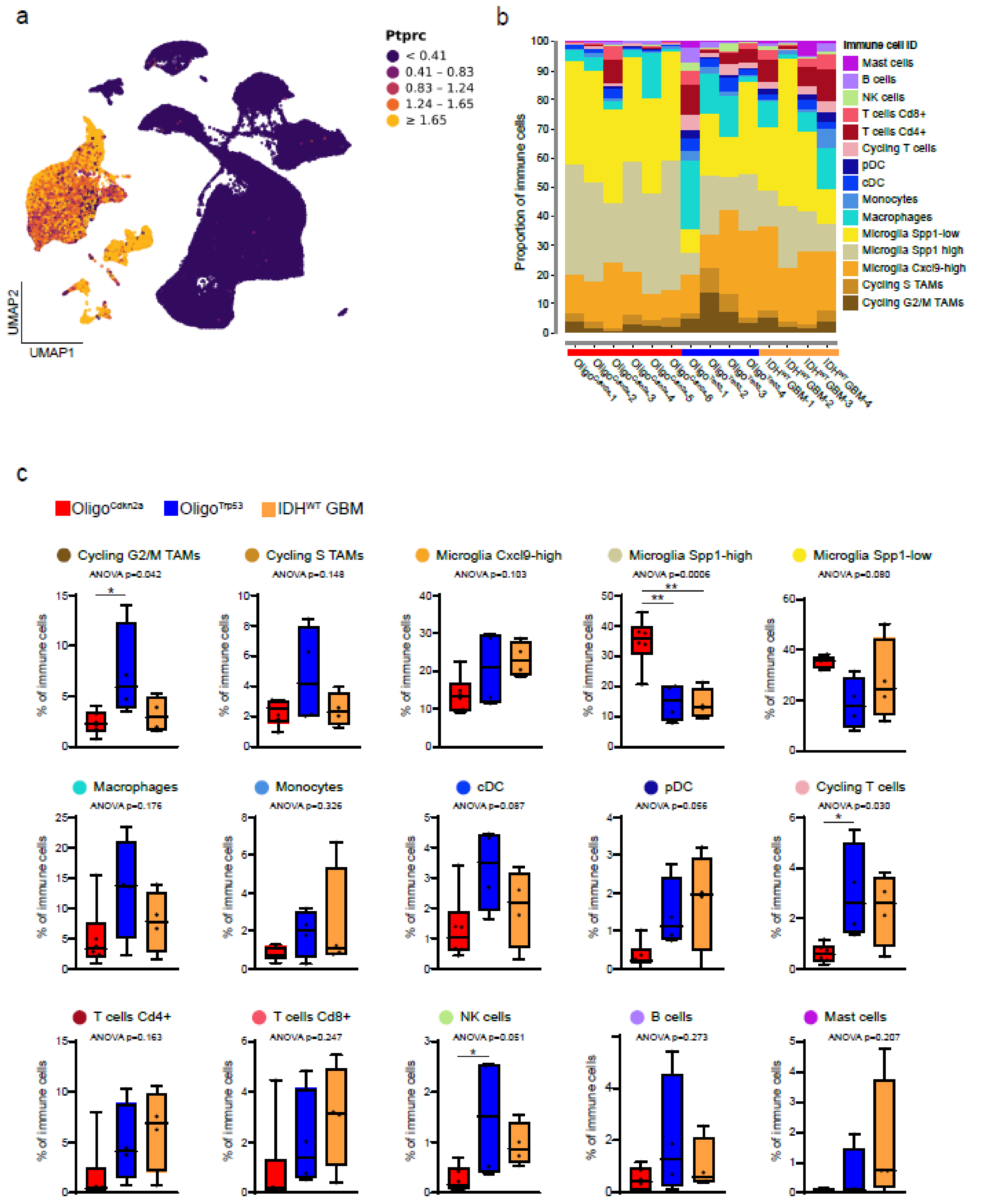

bioRxiv preprint doi: https://doi.org/10.64898/2026.05.14.725183; this version posted May 19, 2026. The copyright holder for this preprint (which was not certified by peer review) is the author/funder, who has granted bioRxiv a license to display the preprint in perpetuity. It is made available under a CC-BY 4.0 International license.

Supplementary Figure 6 – Tumor genotype shapes heterogeneity in the glioma immune microenvironment. a, UMAP visualization of all cells showing expression of *Ptprc* (Cd45+). **b,** Stacked bar plot illustrating the relative proportion of all major cell types in individual tumor samples. **c,** Frequency plots showing the contribution of immune cell-state programs across conditions. Box plots show individual values, median (line), box limits (25th-75th percentiles), and whiskers (min-max). One-way ANOVA p-value displayed, and *p<0.05 or **p<0.01 denotes significance for Tukey's multiple comparisons test. Oligo^{Cdkn2a} n=6, Oligo^{Trp53} n=4, IDH^{WT} GBM n=4. TAMs: tumor associated microglia/macrophages; cDC: conventional dendritic cells; pDC: plasmacytoid dendritic cell; NK: natural killer cell.

bioRxiv preprint doi: https://doi.org/10.64898/2026.05.14.725183; this version posted May 19, 2026. The copyright holder for this preprint (which was not certified by peer review) is the author/funder, who has granted bioRxiv a license to display the preprint in perpetuity. It is made available under a CC-BY 4.0 International license.

Supplementary Figure 7

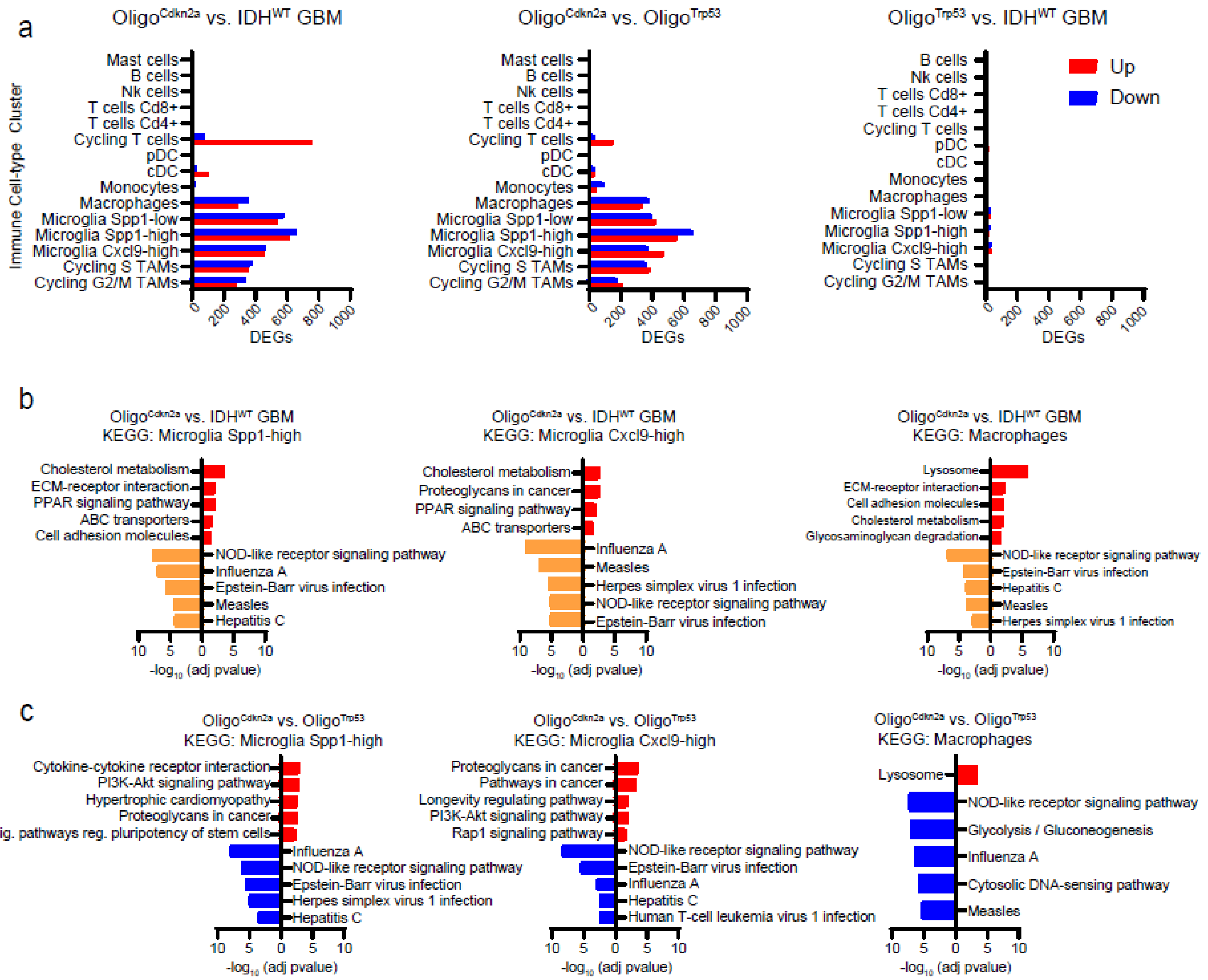

Supplementary Figure 7 – Tumor genotype shapes transcriptional heterogeneity in the glioma immune microenvironment. a, Bar plots showing number of differentially expressed genes (LogFC ≥ 0.7 or ≤ -0.7 and adj p-val < 0.05) identified in pseudo bulk comparisons for each immune cell-type cluster. **b,** Oligo^{Cdkn2a} vs IDH^{WT} GBM comparison, ORA of KEGG gene sets identified based on DEGs in noted immune cell-type cluster. Up to the top five most significant gene sets are plotted by $-log_{10}$(adj-pvalue). **c,** Oligo^{Cdkn2a} vs Oligo^{Trp53} comparison, ORA of KEGG gene sets found in DEGs based on pseudo bulk comparison in noted immune cell-type cluster. Up to the top five most significant gene sets are plotted by $-log_{10}$(adj-pvalue).
